# Nitrate of Silver in Sub-Acute Dysentery

**Published:** 1850-01

**Authors:** Wm. Matthews

**Affiliations:** Eberle, Indiana


### ARTICLE IV.

*The Nitrate of Silver in Sub-Acute Dysentery.*—By W. Matthews, M.D., of Eberle, Indiana.

J. P. L., the subject of the disease, aged 27 years, was of good constitution, and, with the exception of an occasional excess at the table, of temperate habits. Three weeks prior to the date of this report, he was attacked with acute dysentery—the exciting cause apparently being over eating. Although the attack was very severe, the discharges being frequent, slimy and bloody—tormina excruciating, and tenesmus exceedingly distressing, and the stomach rejecting everything taken into it, he had no efficient medical aid for near two weeks. At this time I took charge of the case and treated it on general principles—with calomel and opium, the sugar of lead, and epispastics, but without permanent good effects; (the patient was wretchedly nursed,) and at the expiration of three weeks, the following were the

*Symptoms.*—Pulse, about 80, small and quick; tongue brown and dry, with raw, florid edges; urine, scant and red; skin dry, and the whole surface exsanguineous and shrunken thirst; stomach irritable, with complete anorexia. The stools were frequent, and consisted of a dark brown, jelly-like matter; and they were extremely fetid, or rather, they gave off "a peculiar sickening odor." The patient was racked with tormina and tenesmus. His abdomen was flat and retracted and upon firm pressure being made over the tract of the colon, pain was elicited.

*Diagnosis.*—Extensive Ulceration of the Colon.

*Treatment.*—℞ Nitras Argenti, grs. 2; Opium, grs 8; form into sixteen pills—one to be taken every six hours. When I

next visited the patient, (forty-eight hours afterwards,) nine of the pills had been taken, and I was agreeably disappointed in finding a very manifest improvement was going on—every unfavorable symptom appeared to be slowly yielding. The pulse was fuller, the tongue cleaner and more moist; the stools less frequent, and inclining to be more natural; and the tormina and tenesmus were greatly relieved. The patient expressed himself as feeling "much better." The Nitrate was now discontinued, and the opium directed to be continued.—But, two days after, when I made my next visit, I was mortified at finding the patient relapsed into his former hazardous state. He was again put upon the silver and opium, and this combination, in gradually decreasing doses, was continued for about ten days, at the end of which time I had the satisfaction of being able to pronounce the patient fairly convalescent; and, although badly nursed, he required no farther treatment, but went on gaining strength, until in three weeks he was able to walk abroad.

*Remarks.*—Under the ordinary mode of treating sub-acute dysentery, I feel very confident that this patient would have succumbed. It is to be presumed, that in such cases as the above, the beneficial effects of the nitrate of silver are attributable, first, to its local stimulating action over the indolent ulcerations of the mucous surface; and, secondly, to the genuine tonic influence which it is capable of exercising over the various enfeebled organisms. Although it is not *a new remedy*, having been highly extolled by several eminent physicians in the cure of dysentery, particularly that form which accompanies typhoid fever, (dothinenteritis,) and which depends upon ulceration—it is to be regretted that the nitrate of silver is so much neglected by the great mass of practitioners in the management of the several varieties of sub-acute and chronic lesions of the membrane of the alimentary canal.

